# Implementation of a home-based colorectal cancer screening intervention in Malaysia (CRC-SIM)

**DOI:** 10.1186/s12885-022-10487-6

**Published:** 2023-01-06

**Authors:** Désirée Schliemann, Kogila Ramanathan, Nor Saleha Binti Ibrahim Tamin, Ciaran O’Neill, Christopher R Cardwell, Roshidi Ismail, Zaid Kassim, Frank Kee, Tin Tin Su, Michael Donnelly

**Affiliations:** 1 Centre for Public Health and UKCRC Centre of Excellence for Public Health, Queen’s University Belfast, Belfast, UK; 2 Global Public Health, Jeffrey Cheah School of Medicine and Health Sciences, Monash University Malaysia, Selangor, Malaysia; 3 South East Asia Community Observatory (SEACO), Jeffrey Cheah School of Medicine and Health Sciences, Monash University, Petaling Jaya, Malaysia; 4 Ministry of Health Malaysia, Putrajaya, Malaysia; 5 Segamat District Health Office, Johor, Malaysia

**Keywords:** Colorectal neoplasms, Early detection, Screening, Fecal occult blood test, Implementation Science, LMIC

## Abstract

**Introduction:**

The Colorectal Cancer Screening Intervention for Malaysia (CRC-SIM) was a CRC study of home-based testing designed to improve low screening uptake using the immunochemical fecal occult blood test (iFOBT) in Malaysia.

**Methods:**

This quasi-experimental study was informed by the Implementation Research Logic Model and evaluated with the RE-AIM framework. Trained data collectors recruited by phone, randomly selected, asymptomatic adults aged 50-75 years from Segamat District, who previously completed a health census form for the South East Asia Community Observatory (SEACO). Participants were posted an iFOBT kit and asked to return a photo of the completed test for screening by health care professionals. A regression analysis of evaluation data was conducted to identify which variables were associated with the outcome indicators of ‘study participation’ and ‘iFOBT completion’ and the CRC-SIM was evaluated in terms of its appropriateness, feasibility and acceptability.

**Results:**

Seven hundred forty-seven eligible adults (52%) agreed to participate in this study and received an iFOBT kit. Participation was significantly lower amongst Chinese Malaysians (adjusted OR 0.45, 95% CI 0.35 - 0.59, *p*<0.001) compared to Malays and amongst participants from the rural sub-district (Gemereh) (adjusted OR 0.71, 95% CI 0.54 - 0.92, *p*=0.011) compared to the urban sub-district (Sungai Segamat). Less than half of participants (42%, *n*=311/747) completed the iFOBT. Test-kit completion was significantly higher amongst Chinese Malaysians (adjusted OR 3.15, 95% CI 2.11 - 4.69, *p*<0.001) and lower amongst participants with a monthly household income ≥RM 4,850 (adjusted OR 0.58, 95% CI 0.39 - 0.87, *p*=0.009) compared to participants with a lower household income. The main reported reason for non-participation was ‘not interested’ (58.6%) and main implementation challenges related to invalid photographs from participants and engaging iFOBT positive participants in further clinic consultations and procedures.

**Conclusion:**

Home-testing for CRC (test completion) appeared to be acceptable to only around one-fifth of the target population in Malaysia. However, mindful of the challenging circumstances surrounding the pandemic, the CRC-SIM merits consideration by public health planners as a method of increasing screening in Malaysia, and other low- and middle-income countries.

## Introduction

Screening with the guaiac fecal occult blood test (gFOBT) and immunochemical fecal occult blood test (iFOBT) reduces CRC-related mortality [1, 2]. They are the most commonly used colorectal cancer (CRC) screening tests designed to detect traces of blood in stool samples. In the last decade, high-income countries have introduced home-based gFOBTs/iFOBTs to improve CRC screening accessibility and uptake population-wide [3]. Most low- and middle- income countries (LMICs) do not have the resources to implement population-wide screening and, instead, offer opportunistic screening to people aged ≥40 or ≥50 years [4]. In Malaysia, where CRC is the most common cancer among males and the second most common cancer among females [5], screening guidelines recommend opportunistic screening in clinics using the iFOBT for patients aged 50-75 years [6]. However, uptake is less than 3% amongst the target population [7] and over 70% of CRC cases are detected at stage III or IV [5]. The COVID-19 pandemic placed additional resource constraints on health care systems globally and the phased movement control order (MCO) or lockdown measures that was implemented in Malaysia to stop the spread of the virus, restricted travel for communities to 10km in 2020/2021 [8]. Additionally, health care resources have had to be adapted and redistributed to follow public health guidelines and meet demand for COVID-19 testing and treating the increased number of critically ill patients. Cancer screening, amongst other essential health services, has been impacted negatively and many cancer screening programmes have been paused. Continuing to offer effective cancer screening services during a pandemic is crucial to ensure the successful treatment of most common types of cancer [9]. Due to social distancing rules and travel restrictions, primary care clinics rapidly adapted telehealth communication to assess and support patients. Furthermore, home-testing, the use of smartphones and apps to track health information has been rapidly introduced to reduce the spread of the virus and these ‘technologies’ have quickly become accepted and normalised internationally [10, 11]. In this context, we designed a study of the implementation of home-based CRC screening in order to test its appropriateness, feasibility and acceptability during the COVID-19 pandemic in a LMIC. The primary objective of this study was to describe and evaluate the implementation of the CRC-SIM.

## Methods

This was a quasi-experimental collaborative study conducted by the South East Asia Community Observatory (SEACO) at Monash University Malaysia and Queen’s University Belfast supported by the Ministry of Health Malaysia. The protocol for the **C**olo**r**ectal **C**ancer **S**creening **I**ntervention for **M**alaysia (CRC-SIM) has been registered with the National Medical Research Registry (NMRR) Malyasia (Reference: ID-21-02045-O7G(2); accepted 19/01/2022) and has been submitted for publication [12] and is described here in brief.

### Study population and sampling

SEACO conducted a baseline enumeration (census) in 2012-2013 of five sub-districts of the Segamat District (Bekok, Chaah, Gemereh, Jabi and Sungai Segamat), covering a total population of 44,902 adults with an ethnic representation of Malays, Chinese, Indians and Aborigines [13]. Individuals were revisited for an update interview every one or two years; and every five years, a more detailed health profile is collected on all participants who provided informed consent. The current study drew participants from the 2018 health survey (*n*= 24,710) who previously consented to be included in future studies and meet the following inclusion criteria:

- Males and females aged between 50 and 75 years
- Residents in Sungai Segamat (urban) or Gemereh (rural) sub-districts
- Registered a mobile phone number with SEACO
- Have access to a smartphone and the mobile phone application ‘What’s App’

Participants were excluded if they:

- Reported a history of CRC
- Experienced CRC symptoms at the time of recruitment for this study

### Sampling size and procedure

It was estimated that a sample size of approximately 780 participants would allow the true percentage uptake of screening to be determined with 95% confidence within plus or minus 2.1% (i.e. the 95% confidence interval will span 4.2 percentage points if the uptake is around 90%); and provide a precise estimate of the true percentage uptake of screening by income and ethnic group e.g. in the poorest 50% (based on median monthly income RM 4,850/£720), uptake of screening will be determined within +/- 6%, and, in the Chinese, uptake of screening will be determined within +/- 5%. Random sampling of study participants was applied to select the study population from the SEACO database. We sampled 2829 people to achieve a sample size of 780.

### Intervention

The design of the CRC-SIM was guided by the Implementation Research Logic Model [14] and informed by findings from a scoping review about the implementation and evaluation of CRC screening in LMICs [4] as well as qualitative research with members of the local community [15], health care staff and policy makers in Malaysia. The intervention was delivered between August and November 2021. Eligible members of the SEACO community survey were randomly selected and recruited over the phone by trained SEACO data collectors. Then, a screening pack was sent to participants containing an iFOBT kit, a stool container, an illustrated leaflet about how to collect a stool sample and complete the test, a glove, face covering to mask the smell and a biohazard plastic waste bag. Participants also received a research-informed video through What’s App Messenger App of a medical officer from Klinik Kesihatan Segamat, Ministry of Health Malaysia (a government primary care clinic) explaining the importance of the early detection of CRC, how to collect a stool sample and complete the test, as well as addressing common barriers such as feelings of disgust related to stool collection and fear of test results. Participants were asked to complete the stool test (i.e. one sample of stool) at home and send a photo of the completed test to a confidential SEACO number. Participants received a text message reminder after one week and a phone call after two weeks (from a member of the research team) if they had not shared their completed iFOBT. Two medically trained members of the research team each interpreted the same received completed iFOBT separately and then sent a text to a participant within 10 days if the result was negative or a photograph of a referral letter to Segamat health clinic for further investigation if the test result was positive. Doctors at the clinic who saw iFOBT positive participants referred them to the local hospital where a colonoscopy was scheduled.

### Evaluation

The intervention was evaluated using the RE-AIM outcomes framework (reach, effectiveness, adoption, implementation and maintenance) and measures of acceptability, appropriateness and feasibility [12]. The most recently conducted annual health survey provided socio-demographic and health information about each participant and this information was linked to primary data collection about outcomes that was captured by SEACO. Information about non-completion of an iFOBT kit was collected by research staff during a phone call that they conducted with each participant, two weeks after sending a kit to them.

Post-intervention interviews were conducted with a sub-sample of purposively selected study participants. Participants were selected based on their ethnicity, gender and completion of study components and asked a combination of qualitative and quantitative questions during a telephone interview [12]. Findings from the quantitative response-type questions are described here and the qualitative data is undergoing analysis and will be reported separately.

### Statistical analysis

Data were analysed using SPSS v 24. Descriptive statistics are displayed as frequencies (percentage) for categorical variables and as means and standard deviations (SDs) for continuous variables. Chi-square tests were conducted to compare a) people who agreed to participate in the study vs people who did not agree to participate and b) people who agreed to participate and completed the iFOBT (and sent the photo of the completed test to SEACO) vs people who agreed to participate and did not complete the iFOBT (and did not send the photo of the completed test to SEACO). Logistic regression analyses were conducted in order to identify which variables were associated with the binary outcome measures of ‘participation’ and ‘iFOBT completion’. The analyses were adjusted for key variables (sex, age, ethnicity, education level, employment, household income and history of cancer) as well as variables that were significantly different (or close to significance) between participants and non-participants in the Chi-square test (number of people living in a household, presence of hypertension, smoking history and study sub-district). We reported the mean difference and 95% confidence intervals (95% CI) and odds ratios (OR) and 95% CIs for the main results. More than one participant was recruited from some households, so, the analysis was repeated to adjust for potential clustering within households (as the cluster variable) using robust standard errors using STATA. Adjusting for household clustering did not change the main findings notably and hence are not presented.

## Results

### Reach

A total number of 2,829 eligible adults were selected randomly from the SEACO database to be recruited for this study; 972 people were uncontactable or passed away and 428 people did not meet inclusion criteria when they were contacted, i.e. they did not have What’s App (44.2%) or a smartphone (17.8%), they moved outside the study district (16.8%), experienced signs of CRC at time of recruitment (14.5%), or were aged >75 years (2.6%). Approximately half (52.3%, 95% CI 49.6% to 54.8%) or 747 of the remaining 1,429 people agreed to participate. The most common reasons for non-participation were ‘not interested to join’ (58.6%), feeling healthy and perceived that they did not need the test (10.1%), already attended regular health checks (6.4%), not having time because of work (6%) and ‘worried about COVID-19’ (4.1%). Amongst participants, Malays were the most commonly represented ethnic group (72.3%) followed by Chinese (24%), Indians (2.8%) and other ethnic groups (0.9%). Just over half of participants were, respectively, male (56.5%), aged between 50-59 years (52.5%), were not working (53.5%) and lived in a household of ≥4 people. The majority of participants lived in Sungai Segamat (73.4%), completed either secondary or tertiary education (72.6%), earned a low income, that is, <RM 4,850 (78.6%) and never smoked (63.3%).

Chinese Malaysian community members were significantly less likely to participate compared to Malays (adjusted OR 0.45, 95% CI 0.35 to 0.58, *p*<0.001) (Table 1). The only variable other than ethnicity that was significant after adjustment was study sub-district, i.e. people from Gemereh were less likely to participate in the study compared to people from Sungai Segamat (adjusted OR 0.71, 0.54 to 0.92, *p*=0.011). Participation appeared to be highest among the youngest age category (50-54 years old) but after adjustment the difference between age groups was attenuated (Table 1). Similarly, people who completed varying levels of education vs no formal education seemed more likely to participate but this was also attenuated after adjustment (Table 1). The same pattern was repeated for people in households of four to five family members or ≥6 family members who seemed more likely to participate than smaller households but not after adjustment.

**Table 1:** The relationship between the socio-demographic characteristics of respondents and study participation

	*N* people agreed to participate	*N* people eligible	*%*	*P*^*a*^	OR (95% CI) (unadj)	*P*	OR (95% CI) (adj)^b^	*P*
**Age**								
50-54	216	363	59.5	0.025	*1.00 (Reference)*		*1.00 (Reference)*	
55-59	174	346	50.3)		0.69 (0.51; 0.93)	0.014	0.77 (0.56; 1.05)	0.102
60-64	183	358	51.1		0.71 (0.53; 0.96)	0.024	0.89 (0.64; 1.24)	0.500
65-69	117	239	49.0		0.65 (0.47; 0.91)	0.011	0.91 (0.63; 1.33)	0.636
70 and above	57	123	46.3		0.59 (0.39; 0.89)	0.011	1.00 (0.74; 1.36)	0.950
**Gender**								
Male	422	781	54.0	0.144	*1.00 (Reference)*		*1.00 (Reference)*	
Female	325	648	50.2		0.86 (0.70; 1.06)	0.144	1.00 (0.74; 1.36)	0.997
**Ethnicity**								
Malay	540	923	58.5	<0.001	*1.00 (Reference)*		*1.00 (Reference)*	
Chinese	179	461	38.8		0.45 (0.358; 0.57)	<0.001	0.45 (0.35; 0.58)	<0.001
Indian	21	32	65.6		1.35 (0.645; 2.84)	0.423	1.39 (0.65; 2.96)	0.392
Others	7	13	53.8		0.83 (0.276; 2.48)	0.735	0.99 (0.31; 3.18)	0.984
**Education**								
No formal education	15	38	39.5	<0.001	*1.00 (Reference)*		*1.00 (Reference)*	
Primary	179	413	43.3		1.17 (0.60; 2.3)	0.645	1.14 (0.57; 2.29)	0.718
Secondary	465	825	56.4		1.98 (1.02; 3.85)	0.044	1.66 (0.82; 3.34)	0.156
Tertiary	77	135	57.0		2.04 (0.98; 4.24)	0.058	1.69 (0.78; 3.65)	0.183
**Working status**								
Not working	400	785	51.0	0.283	*1.00 (Reference)*		*1.00 (Reference)*	
Working	346	643	53.8		1.12 (0.91; 1.38)	0.283	1.02 (0.79; 1.31)	0.904
**Household income**								
<RM 4,850 (low)	587	1141	54.4	0.212	*1.00 (Reference)*		*1.00 (Reference)*	
≥RM 4,850	160	288	55.6		1.18 (0.91; 1.53)	0.913	1.18 (0.89; 1.56)	0.248
**Household size**								
Living alone	27	66	40.9	0.032	*1.00 (Reference)*		*1.00 (Reference)*	
2 to 3	338	679	49.8		1.43 (0.86; 2.39)	0.171	1.30 (0.77; 2.21)	0.332
4 to 5	254	450	56.4		1.87 (1.11; 3.16)	0.019	1.45 (0.84; 2.50)	0.185
≥6	128	234	54.7		1.74 (1.00; 3.04)	0.049	1.44 (0.81; 2.57)	0.217
**Cancer history**								
Yes	6	13	46.2	0.655	*1.00 (Reference)*		*1.00 (Reference)*	
No	737	1407	52.4		1.28 (0.43; 3.84)	0.655	0.72 (0.23; 2.25)	0.576
**Study sub-district**								
Sungai Segamat	548	1052	52.1	0.817	*1.00 (Reference)*		*1.00 (Reference)*	
Gemereh	199	377	52.8		1.03 (0.81; 1.30)	0.817	0.71 (0.54; 0.92)	0.011
**Hypertension**								
Yes	313	630	49.7	0.082	*1.00 (Reference)*		*1.00 (Reference)*	
No	434	799	54.3		1.20 (0.98; 1.48)	0.082	1.17 (0.94; 1.47)	0.160
**Ever smoked**								
Yes	274	487	56.3	0.03	*1.00 (Reference)*		*1.00 (Reference)*	
No	473	942	50.2		0.78 (0.63; 0.98)	0.03	0.84 (0.62; 1.14)	0.257

^a^ Results from the Chi-square test

^b^ Adjusted for age, gender, ethnicity, education, working status, monthly household income, cancer history, study sub-district, hypertension and smoking history

### Effectiveness and adoption

**Fig. 1:**
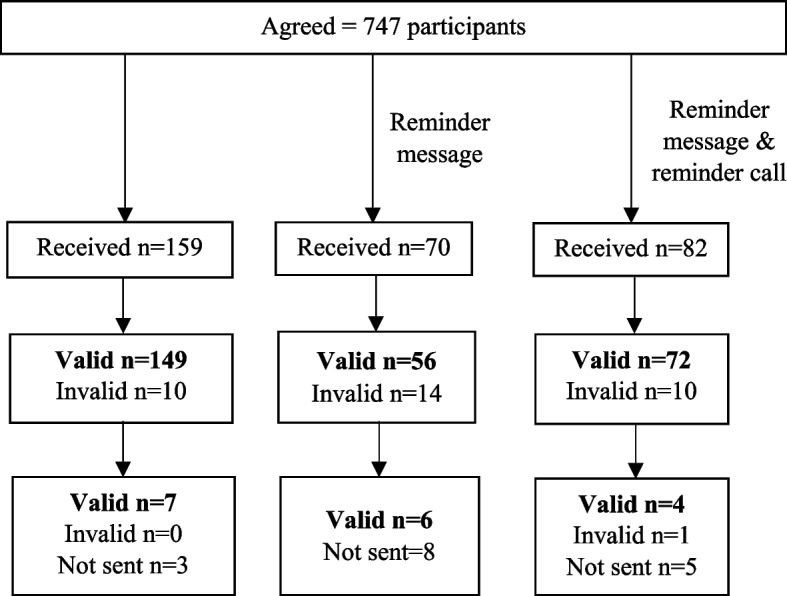
Number of valid/invalid iFOBT results received before and after the message and call reminders

Overall, 311 out of 747 (41.6% 95% CI 38.1% to 45.3%) participants who agreed to participate and who received a testing kit at home, completed a stool test and sent a photo of the completed test to SEACO. Figure 1 presents the number of people who sent the completed test photo without receiving any reminder (159/311, 51%), after a text message reminder (70/311, 23%) and after a phone call reminder (82/311, 26%). A small number of completed and returned iFOBT test photos were invalid (34/311, 11%) and, so, these participants received a second test kit. In total, we received 294 valid test results (95%) from participants. Chinese participants were significantly more likely to complete the test compared to Malays (adjusted OR 3.22, 95% CI 2.17 to 4.79, *p*<0.001) (Table 2). Also, participants with a household income ≥RM 4,850 were less likely to complete the test compared to those with a household income <RM 4,850 (adjusted OR 0.58, 95% CI 0.39 to 0.87, *p*=0.009) (Table 2). Participants who completed tertiary education seemed to be more likely to complete the iFOBT compared to participants without formal education albeit not statistically significantly so (adjusted OR 2.85, 95% CI 0.87 to 9.36, *p*=0.085). The odds of completing the test seemed to be lower amongst participants aged ≥70 years compared to participant aged 50-54 years but this was also not statistically significant (adjusted OR 0.50, 95% CI 0.24; 1.07, *p*=0.073).

**Table 2:** The relationship between the socio-demographic characteristics of respondents and iFOBT completion

	*N* participants who completed the iFOBT	*N* people agreed to participate	*%*	*P*^*a*^	OR (95% CI) (unadj)	*P*	OR (95% CI) (adj)^b^	*P*
**Age**								
50-54	96	216	44.4	0.484	*1.00 (Reference)*		*1.00 (Reference)*	
55-59	75	174	43.1		0.95 (0.63; 1.42)	0.791	0.95 (0.61; 1.48)	0.822
60-64	73	183	39.9		0.83 (0.56; 1.24)	0.359	0.94 (0.59; 1.50)	0.804
65-69	49	117	41.9		0.90 (0.57; 1.42)	0.652	1.01 (0.59; 1.72)	0.985
70 and above	18	57	31.6		0.58 (0.31; 1.07)	0.082	0.50 (0.24; 1.07)	0.073
**Gender**								
Male	169	422	40.0	0.316	*1.00 (Reference)*		*1.00 (Reference)*	
Female	142	325	43.7		1.16 (0.87; 1.56)	0.317	1.41 (0.90; 2.22)	0.136
**Ethnicity**								
Malay	197	540	36.5	<0.001	*1.00 (Reference)*		*1.00 (Reference)*	
Chinese	105	179	58.7		2.47 (1.75; 3.49)	<0.001	3.22 (2.17; 4.79)	<0.001
Indian	8	21	38.1		1.07 (0.44; 2.63)	0.880	1.34 (0.53; 3.41)	0.536
Others	1	7	14.3		0.29 (0.04; 2.43)	0.254	0.37 (0.04; 3.13)	0.359
**Education**								
No formal education	6	15	40.0	<0.001	*1.00 (Reference)*		*1.00 (Reference)*	
Primary	58	179	32.4		0.72 (0.24; 2.12)	0.549	0.70 (0.23; 2.14)	0.529
Secondary	192	465	41.3		1.06 (0.37; 3.01)	0.920	1.13 (0.35; 3.24)	0.826
Tertiary	47	77	61.0		2.35 (0.76; 7.28)	0.138	2.85 (0.87; 9.36)	0.085
**Working status**								
Not working	163	400	40.8	0.631	*1.00 (Reference)*		*1.00 (Reference)*	
Working	147	346	42.5		1.07 (0.80; 1.44)	0.631	0.99 (0.69; 1.44)	0.976
**Household income**								
<RM 4,850 (low)	255	587	43.4	0.055	*1.00 (Reference)*		*1.00 (Reference)*	
≥RM 4,850	56	160	35.0		0.70 (0.49; 1.01)	0.056	0.58 (0.39; 0.87)	0.009
**Household size**								
Living alone	10	27	37.0	0.298	*1.00 (Reference)*		*1.00 (Reference)*	
2 to 3	139	338	41.1		1.19 (0.53; 2.67)	0.678	1.53 (0.63; 3.71)	0.350
4 to 5	116	254	45.7		1.43 (0.63; 3.24)	0.393	1.98 (0.80; 4.92)	0.141
≥6	46	128	35.9		0.95 (0.40; 2.26)	0.914	1.27 (0.49; 3.26)	0.627
**Cancer history**								
Yes	4	6	66.7	0.213	*1.00 (Reference)*		*1.00 (Reference)*	
No	306	737	41.5		0.36 (0.07, 1.95)	0.233	0.34 (0.05; 2.08)	0.241
**Study sub-district**								
Sungai Segamat	233	548	42.5	0.415	*1.00 (Reference)*		*1.00 (Reference)*	
Gemereh	78	199	39.2		0.87 (0.63; 1.21)	0.416	1.09 (0.75; 1.59)	0.652
**Hypertension**								
Yes	135	313	43.1	0.481	*1.00 (Reference)*		*1.00 (Reference)*	
No	176	434	40.6		0.90 (0.67; 1.21)	0.481	0.82 (0.59; 1.14)	0.652
**Ever smoked**								
Yes	106	274	38.7	0.214	*1.00 (Reference)*		*1.00 (Reference)*	
No	205	473	43.3		1.21 (0.90; 1.64)	0.214	0.87 (0.56; 1.36)	0.536

^a^ Results from the Chi-square test

b Adjusted for age, gender, ethnicity, education, working status, monthly household income, cancer history, study sub-district, hypertension and smoking history

Only a few participants gave reasons for non-completion. For example, 97/436 participants (22.2%) changed their mind after they received a test kit and 24/436 (5.5%) reported that collecting a stool sample made them feel nauseous. Almost one-fifth (19%; 56/294) had a positive iFOBT result and were referred to Segamat Health Clinic. 30/56 positive participants visited Segamat Health clinic and 9/31 participants refused to attend a colonoscopy appointment (three participants completed a second iFOBT at the clinic which was negative, four participants were worried about the colonoscopy being painful and another two participants felt healthy and did not think that they needed a colonoscopy). The most commonly reported reasons for not availing of an appointment with a doctor at the clinic were ‘work or family commitments’ (*n*=11), ‘feeling healthy, no bowel issues’ (*n*=10), and fear of colonoscopy procedure or outcome (*n*=4). The colonoscopy procedure was completed for 15 participants at Segamat Hospital and for six participants at a private hospital. Participants attended a private hospital because they had medical insurance or funds that afforded the option of doing so. The outcomes of the colonoscopy investigative procedure were as follows: normal (*n*=10), CRC (*n*=1), polyp (*n*=4), haemorrhoids (*n*=2), diverticular disease (*n*=2) and two participants were further referred for a barium enema.

In the context of this study, it is important to note that the data collectors played a role in the implementation and adoption of the uptake intervention in terms of recruiting participants, processing the arrangements for the delivery of home screening test kits and following-up participants. Experienced SEACO staff trained five field workers (4 Malay, 1 Chinese) over two half-days via Zoom to collect relevant data according to the procedures set out in the study protocol and approved by the Ethics Committee. Training included an introduction to the study and focused on the development of skills regarding interviewing and calling scripts using didactic methods and role play as well as following procedures for data recording and assuring data quality. All data collectors stayed for the full duration of the study, except for the Chinese data collector who left in October.

### Implementation

Recruitment started in August and ended in October 2021. The last iFOBT photo was returned to SEACO in November 2021. On average, it took 2.7 (SD 2.4) days for participants to receive a screening pack after recruitment and about 11.8 (SD 12.2) days for participants to send a photo of the completed test after they received the stool test kit. Recruitment calls lasted 17:14 (mean) (SD 3:24) minutes. It took up to ten days between the time point when a participant sent a photo and SEACO replied with the result. iFOBT positive participants were referred to their local clinic doctor who, in turn, referred them to a general surgeon who scheduled participants for a colonoscopy. Waiting times between seeing a clinic doctor and attending an appointment with a specialist at hospital were three to four weeks; and four to six weeks between an appointment with a specialist and undergoing a colonoscopy procedure. Challenges that were encountered in the implementation of the intervention and any related changes that were made are reported in Table 3.

**Table 3:** Challenges encountered and changes made to the implementation of the intervention

**Intervention component**	**Challenges encountered**	**Changes made to intervention implementation**
Call participant up to 3 times if no answer during recruitment call (on different days and times)	34% of participants selected for this study were uncontactable	A text message was sent after the third attempt to explain that this number belongs to SEACO and the purpose of the call. If people were interested to participate in this study, we encouraged them to let us know a time that suits them or to give us a call back. *Outcome:* None of the participants replied to the text message and this did not make a different to the study
iFOBT home screening test	Female participant completed test during menstruation	*(Solution for participant: Resent the screening pack and asked participant to complete a second test)* Female participants were informed to complete the test when not menstruating during the recruitment call.
Taking photo of the test result and send it to SEACO through What’s App	Some participants required help with taking and/or sending the photo through What’s App	During the reminder call, data collectors asked participants whether they require help/ more information to take/ send the photo and were talked through the steps if needed.
Informing participants of their test result within 7 days	It was not always feasible to provide feedback within 7 days for the medical team members who were asked to review the results.	Extended from 7 to 10 working days and revised in the acknowledgement text message script as well.
Informing participants of their positive result	Some people with a positive iFOBT result did not answer the phone when we called them to inform participant of the result	Participants were called up to three times (different day/time). A voice message was sent to those that didn’t answer and a referral letter with the positive result and referral to doctor.
Invalid results	Some participants sent iFOBTs that showed invalid test results.	Participants who sent the invalid result were called and sent another screening pack (1x) if they agreed to complete the test a second time. The data collectors clarified the procedure again over the phone and answered any questions. If the second iFOBT test was still invalid, then the result was noted as invalid and the participant was referred to the nearest health clinic if they were interested to redo the iFOBT.
Interpreting the result	Some pictures showed very faint lines on the test result	The research staff interpreting results contacted laboratory staff for a third opinion.

### Acceptability

As noted above, quantitative data indicated that the CRC-SIM was acceptable for about half of the eligible participants who agreed to receive and complete the test. In addition, we conducted qualitative interviews with 48 study participants . Table 4 presents the specific views of this subsample of study participants (*n*=48) regarding the acceptability of different study components and the preferences for future contact. Only around half the sample reported that it was acceptable to ask people to complete a stool test at home even with the provision of guidance via video and leaflet. Regarding CRC-screening in the future, participants chose multiple options - phone appeared to be the preferred mode of contact by participants (79.2%) compared to a text message (41.7%), letter (41.7%), a home test-kit (37.5%), a face-to-face invitation (37.5%) or a video invitation (14%).

**Table 4:** Acceptability of study components (*n*=48)

	**N/ d**	**%**
How helpful was the instruction leaflet in describing how to complete the stool test? (helpful/ very helpful)	28/45	62.2
How helpful was the video in describing how to complete the stool test? (helpful/ very helpful)	31/40	77.5
How easy was it for you to collect the stool? (easy/ very easy)	30/43	69.8
How easy was it for you to carry out the stool test at home?	29/45	64.4
How acceptable is it to ask people to complete the stool test at home with the guidance of the video and leaflet? (acceptable/ very acceptable)	25/46	54.3
How helpful was the text message reminder in reminding you to complete the stool test? (helpful/ very helpful)	10/21	47.6
How helpful was the telephone call reminder in reminding you to complete the stool test? (helpful/ very helpful)	5/15	33.3
How easy was it for you to send the photo of the test to SEACO? (easy/ very easy)	31/37	83.8
Was it acceptable to receive the normal test result through text message? (acceptable/ very acceptable)	23/25	92.0
Was it acceptable to receive the abnormal test result over the phone? (acceptable/ very acceptable)	22/23	95.7
How easy was it to get an appointment to discuss the results with your doctor? (easy/ very easy)	14/15	93.3
How easy was it to get a referral for a colonoscopy? (easy/ very easy)	12/14	85.7
How would you rate your overall satisfaction with the colorectal cancer screening study? (satisfied/ very satisfied)	38/48	79.2

d – number of participants who this question was applicable to

### Feasibility

Overall, and mindful of the pandemic context, the results suggest that it was feasible to implement the intervention in the rural and semi-rural communities of Segamat with significant input from SEACO staff. The results indicate that the feasibility of implementing the intervention or service elsewhere in Malaysia would require support staff like the administrative staff at SEACO, particularly for work tasks such as sending reminder messages and storing test photos.

## Discussion

This was the first study in Malaysia and South East Asia to implement a home-based CRC screening test. Our findings suggest that 52% of the target population agreed to participate in the self-administered iFOBT and 42% of those who agreed to participate completed the test and forwarded a photo of the completed test to SEACO (i.e. 21% overall uptake). According to European guidelines for CRC screening, the lowest acceptable uptake of screening is 45% [16]. However, it is important to note that these guidelines apply to population-based CRC screening and are based on the experience of screening programmes that have been implemented for a number of years in most European countries, whereas the screening intervention in this study was the first of its kind to be implemented in Malaysia. Also, health literacy, i.e. “the skills that enable individuals to obtain, understand, appraise and use information to make decisions and take actions that will have an impact on health status” [17] is likely to be lower in LMICs where socio-economic hardship and illiteracy, factors associated with low health literacy [18], are more prevalent compared to high-income countries. Malaysians overall health literacy level has previously been categorised at a lower sufficiency level [19], which might partly explain the relatively low screening uptake.

Findings from a recent review of studies about CRC screening in LMICs suggested that FOBT/ iFOBT completion ranged between 14% and 98% [4] and screening in all studies except one were conducted face-to-face, in community clinics. The only other study in a LMIC that used self-testing at home was conducted in Bulgaria in 2013 - general practitioners invited health-insured patients, aged ≥45 years who had at least one consultation in the previous year, to participate in home-testing [20]. Participation rate (including initial agreement and subsequent completion of an iFOBT) was 79% and was significantly higher amongst females compared to males and those aged 45-54 compared to older age groups. It is probable that the higher participation rate was due to mode of recruitment, i.e. a personal invitation (through call or e-mail) to only 20 patients per GP from their usual GP with whom they had been in contact at least once in the previous year vs a phone call from a dedicated SEACO research team member. Also, participants collected the home screening test kit from their GP who answered any questions that patients might have had about the screening and generally provided reassurance. The study reported lower participation amongst males, older age groups and participants from villages compared to urban areas. This pattern of results was similar to findings from our study – participants aged ≥70 years seemed less likely to complete a iFOBT compared to younger age categories (e.g. 50 to 54 years), although after adjustment this was not significant, and participants from villages (i.e. the sub-district Gemereh) were less likely to agree to participate compared to residents of the town area, Sungai Segamat. A reason for lower uptake amongst older age groups may be technical in nature related to the completion of an iFOBT [20].

Significant differences between ethnic groups were noted in our study. Chinese Malaysians were less likely to participate compared to Malays, but significantly more likely to complete the iFOBT. In our previous studies, Chinese Malaysians appeared to be less aware of cancer symptoms than Malays [21, 22] and more likely to delay seeking help for symptoms that perhaps were perceived to be less salient such as a persistent cough but more likely than Malays to seek help for arguably clearer signs of potential health problems such as rectal bleeding [23]. Chinese Malaysians are more likely to have private medical insurance and to engage in regular medical check-ups [24], perhaps, they rely on this arrangement to identify symptoms of concern and only initiate help-seeking when they perceive a serious symptom. How these attitudes and behaviours might contribute to explaining our finding about differences between Chinese Malaysians and Malays regarding participation and completion of screening, is unclear. Furthermore, participants from the PeKa B40 category (i.e. citizens in the bottom 40% household income range) were significantly more likely to complete the screening test compared to those with a higher household income. This is different to previous findings that suggest that socio-economic deprivation is associated with poor cancer screening perceptions [25] and lower screening uptake [26, 27]. One possible explanation is that participants on a low household income are less likely to have health insurance [25] and are therefore more likely to participate in a screening test free of charge to avoid associated costs, compared to higher income earners.

The findings suggested that participants with secondary or tertiary education may be more likely to participate in an intervention and complete a test which may be linked to better health literacy [28]. Disgust related to collecting a stool sample has been reported previously and consistently across countries as one of the main reasons for non-participation in FOBT/iFOBT screening, as well as fear of finding cancer [29–31] and these reasons again were reported in this study.

Less than half of participants with a positive iFOBT went for a doctor’s appointment after they received the result and even fewer participants went for a follow-up colonoscopy, which is an issue that has been reported by other CRC studies [4]. A colonoscopy is an invasive procedure but it is important for confirming a CRC diagnosis and initiating treatment. Little can be done to diagnose and treat CRC if participants do not attend their colonoscopy appointment - iFOBT screening is promoted for this reason, that is, to detect potential signs of CRC as early as possible and nudge people with signs towards agreeing to undergo a colonoscopy. Reasons for missing colonoscopies in the UK have been described previously as having other priorities/commitments, an unwillingness to undertake the procedure and thinking that an iFOBT produced a false positive result [32]. It is difficult to draw clear conclusions about these reasons given the lack of reported details in the study. A study in Holland found that nonattendance for a colonoscopy was due to reasons such as a perception of low risk for CRC, aversion and fear of colonoscopy, distrust and a reluctance towards cancer treatment) [33]. Some of these reasons noted above are similar to reasons provided by participants in this study such as (family) commitments and feeling healthy, whereas others, for example, fear of the colonoscopy procedure or of the outcome differed. Furthermore, the MCO implemented by the Malaysian government in response to the COVID-19 pandemic may have contributed to the low clinic and hospital attendance by iFOBT positive participants.

Findings from this study suggest that a CRC home-testing intervention may be acceptable to about 21% of the population in Malaysia, more so amongst people with secondary or tertiary education, younger age groups, lower incomes and town area residents. Different strategies based on, for example, socio-demographic characteristics and health literacy level, may need to be implemented in order to encourage and engage people to participate in iFOBT screening (in a home-testing format or at local health clinics). In addition, there may be a need to educate people about the benefits of early detection and to address fear about cancer screening. There is evidence to suggest that telephone contact regarding for example the provision of additional clear instructions and reminders, advanced notification about screening, GP endorsement letters and telephone contact as well as simplified test procedures are likely to improve uptake of mail-out CRC screening programmes [34]. The administrative load was high for the implementation and delivery of this intervention. However, automation, for example, of reminders and storing of test photos are likely to reduce the burden of such tasks and increase feasibility. Future research needs to explore how home test participation and completion might be encouraged further and how administrative and tasks might be reduced.

This study identified and recruited participants through SEACO which holds socio-demographic data and contact information about Segamat residents who agreed that they could be contacted to participate in future research and that their data could be used to investigate health issues such as low uptake of screening services. SEACO is particular to the Segamat District and, so, there would be a need to identify an alternative way of obtaining this kind of information about eligible participants (perhaps via patient lists at local health clinics or other government-owned databases) if there was a plan to rollout targeted home-based testing in order to increase uptake. A limitation of this study is that only one clinic was involved in the study for the follow-up appointments and the implementation data reported here is therefore limited. Furthermore, we did not have data about the proportion of eligible study participants who declined participation. Also, we did not conduct a power calculation with respect to making between group differences and this point should be noted when interpreting the results. It is important, too, to keep in mind the quasi-experimental nature of the study design when considering the findings.

## Conclusion

Arguably, the CRC-SIM study is the first of its kind to test CRC home-screening in South East Asia. The findings indicate that CRC home-testing is an acceptable way to screen for CRC in Segamat district and should be considered as either an alternative option to screening at clinics or to supplement current practices. We have reported a number of implementation challenges and consideration for future CRC screening programmes. Further research is required to test the implementation of home-testing outside of Segamat district to allow us to make recommendations about a potentially nationwide roll-out.

## Data Availability

The datasets used and/or analysed during the current study are available from the corresponding author or the SEACO website (https://www.monash.edu.my/seaco/research-and-training/how-to-collaborate-with-seaco) on reasonable request.

## Abbreviations

CI: Confidence interval
CRC: Colorectal Cancer
CRC-SIM: Colorectal Cancer Screening in Malaysia
gFOBT: Guaiac fecal occult blood test
iFOBT: Immunochemical fecal occult blood test
LMIC: Low- and middle- income country
N: Number
OR: Odds ratio
SEACO: South East Asia Community Observatory
SD: Standard deviation
